# Aberrant Expression of ID2 protein and its correlation with EBV-LMP1 and P16(INK4A) in Classical Hodgkin Lymphoma in China

**DOI:** 10.1186/1471-2407-8-379

**Published:** 2008-12-19

**Authors:** Po Zhao, Yali Lu, Lin Liu, Mei Zhong

**Affiliations:** 1 Department of Pathology, Chinese People's Liberation Army (PLA) General Hospital, 28 Fuxing Road, Beijing 100853, PR China

## Abstract

**Background:**

The relationships between the expression of ID2, EBV-LMP1 and P16(INK4A) in Chinese classical Hodgkin lymphoma are unknown and need exploring.

**Methods:**

Samples of classical Hodgkin lymphoma from 60 Chinese patients were analyzed for the expression of ID2, EBV-LMP1 and p16(INK4A) proteins by immunohistochemistry.

**Results:**

ID2 protein was expressed in 83.3% of this group of classical Hodgkin lymphoma, staining strongly in both cytoplasm and nucleus of the Hodgkin and Reed-Sternberg (HRS) cells. EBV-LMP1 and P16(INK4A) were overexpressed in 85.0% and 71.7% of Hodgkin lymphoma, respectively. EBV-LMP1 was noted in the cytoplasm, membrane and nucleus of HRS cells; P16(INK4A) was in the nucleus and cytoplasm. Microscopically, ID2, EBV-LMP1 and P16(INK4A) staining distinguished the HRS cells from the complex background of lymphocytes. ID2 was positively correlated with EBV-LMP1(*P *< 0.01), but P16(INK4A) was inversely related to EBV-LMP1 (*P *< 0.05).

**Conclusion:**

It is suggested that ID2, EBV-LMP1 and P16(INK4A) could play an important role in the evolution of classical Hodgkin lymphoma, and be considered as potential adjunct markers to identify HRS cells in diagnosis.

## Background

Hodgkin lymphoma comprises two distinct entities; namely nodular lymphocyte predominant Hodgkin lymphoma and classical Hodgkin lymphoma. Classical Hodgkin lymphoma is a lymphoid neoplasm composed of monoclonal Hodgkin cells and multinucleated Reed-Sternberg (HRS) cells residing in an infiltrate containing a variable mixture of non-neoplastic small lymphocytes, eosinophils, neutrophils, histiocytes, plasma cells, fibroblasts and collagen fibres. [1] There are four morphological variants of classical Hodgkin lymphoma, namely nodular sclerosis, mixed cellularity, lymphocyte-rich and lymphocyte-depleted. HRS cells are currently thought to be derived from mature B cells at the germinal stage of differentiation, and are pathognomonic in Hodgkin lymphoma. [1] However, HRS cells may often be scanty and difficult to identify in sections. The pathogenesis of classical Hodgkin lymphoma remains unknown.

ID proteins are key regulators in several developmental and cellular processes. In general, aberrant ID expression seems to favor proliferation, to inhibit differentiation, and to facilitate tumor neoangiogenesis.[2] From early B-cell development to mature B cells, E2A is the first one of the three transcription factors (E2A, EBF, and PAX5) to be expressed, and E2A together with EBF regulates the expression of PAX5.[3,4] The inhibitor of DNA binding ID2 (inhibitor of DNA binding 2 or inhibitor of differentiation, ID2), may bind and negatively regulate E2A and PAX5[5,6] by direct interaction. ID2 expression in developing hematopoietic cells seems to repress B-cell development and B-cell-specific gene expression and to favor development of other lineages, [7-11] whereas in mature B cells, ID2 is up-regulated during plasma cell differentiation with concomitant loss of expression of several B-cell genes.[12] Furthermore, the balance between ID2 and E2A, and between ID2 and PAX5 seem to be important for B-cell differentiation.[13,14] ID2 has been found strongly and uniformly expressed in the HRS cells of classic Hodgkin Lymphoma and probably represses B-cell-specific gene expression by inactivation of E2A (and perhaps also PAX5). [14] Expression of two other members of the ID (inhibitor of differentiation) family of proteins, ID1 and ID3, was found to be induced in the presence of latent membrane protein 1 (LMP1), the Epstein-Barr virus oncoprotein, by the activation of NF-kappaB, phosphatidylinositol 3-kinase, mitogen-activated protein kinase, and c-Jun N-terminal kinase signaling[15,16]. However, there is to date no study on the relationship between ID2 and EBV-LMP1 in Hodgkin lymphoma. Overexpression of P16(INK4A)[17] has been used as a diagnostic adjunct in premalignant and malignant humanpapilloma virus (HPV) lesions in gynecologic pathology[17] in recent years. Previous research [18] also suggested that EBV-LMP1 and P16(INK4A) had a role in the carcinogenesis of classical Hodgkin lymphoma, but there was no corresponding investigation of associations between ID2, EBV-LMP1 and p16(INK4A), which might provide further understanding of the mechanism. Therefore, additional studies are needed to identify whether ID2, EBV-LMP1 and P16(INK4A) have similar diagnostic value and to explore the possible relationship between them in classical Hodgkin lymphoma. We used the ID2, EBV-LMP1 and P16(INK4A) antibodies to investigate the possible role of the expression of the three proteins in 60 cases of Chinese classical Hodgkin lymphoma. It is hoped that the study will give information on the pathogenesis of this disease.

## Methods

### Patients

Sixty patients (36 men, 24 women) with histologically confirmed classical Hodgkin lymphoma were recruited into the study. Ethical approval for this study was not required by our institute as the experiments carried out did not relate to patient's privacy, impairment or treatment. The Hodgkin lymphomas were classified as nodular sclerosis (n = 29), mixed cellularity (n = 11), lymphocyte-rich (n = 16) and lymphocyte-depleted (n = 4) variants according to the World Health Organization recommendations [1].

### Immunohistochemistry

A formalin-fixed, paraffin-embedded block was selected from each patient with Hodgkin lymphoma for immunohistochemical study. Paraffin sections, 4 μm in thickness, were cut, dewaxed in xylene and rehydrated in a graded ethanol series. Then sections were immersed in 3% hydrogen peroxide in methanol for 10 minutes to block endogenous peroxidase activity and rinsed in running water. Subsequently they were immersed in boiling 0.01 M citrate buffer (pH 6.0) in a pressure cooker, which was then sealed and brought to full pressure for 2 minutes. It was then de-pressured and cooled under running water. The lid was then removed, and the hot buffer was flushed out with cold water from a running tap. The cooled sections were washed twice in phosphate buffered saline (PBS) before immunocytochemical staining.

The sections were then covered the primary antibodies. These polyclonal rabbit antibody against human ID2 protein (Santa Cruz, CA, USA), mouse monoclonal antibody to Latent membrane protein 1 of the Epstein-Barr virus (EBV-LMP1) (CS.1–4, DAKO) and mouse monoclonal antibody against human P16(INK4A) protein (Zymed, South San Franciso, CA, USA), all diluted 1 in 100. After exposure to primary antibody for 1 hour, the sections were allowed to react with poly peroxidase-anti-mouse/rabbit IgG for 20 minutes by the standard PV-6000 Polymer Detection System (Zymed, South San Franciso, CA, USA). The system is a non-biotin detection system, thus, avoiding non-specific staining due to endogenous biotin. The sections were then washed in water, counter-stained with Mayer's haematoxylin for one minute at room temperature, dehydrated, cleared and mounted.

Human cervical squamous cell carcinoma tissues were used as a positive control for P16(INK4A), smooth muscle cells of vessels were used as an internal positive control for ID2 and human nasopharyngeal carcinoma was used as a positive control for EBV-LMP1. Negative controls were sections treated as above but with the primary antibody replaced by 0.01 M PBS. Expression of P16(INK4A), ID2 and EBV-LMP1 was considered positive when at least 10% of HRS cells were stained. In addition, 10 B-cell lymphomas, 8 T-cell lymphomas and 10 reactive lymph nodes (4 lymph nodes sampled during resection of carcinomas and 6 lymphadenitis) were used for comparison. The B-cell lymphomas comprised 5 diffuse large B-cell lymphomas, 4 extranodal marginal zone B-cell lymphomas (MALT lymphomas) and 1 Burkitt lymphoma. The T-cell lymphomas were 2 anaplastic large cell lymphomas, 2 extranodal NK/T-cell lymphomas of nasal type, 2 angioimmunoblastic T-cell lymphomas and 2 peripheral T-cell lymphomas, unspecified.

### Statistical analysis

The correlation between expression of the proteins was determined by counting the positive cells in at least four medium power fields of serial sections, calculating and presenting as means ± SD. The statistical significance of association was assessed by Spearman's correlation coefficient test, with SPSS15.0. A value of *P *less than 0.05 was accepted as statistically significant.

## Results

### Expressions of ID2, EBV-LMP1 and P16(INK4A) in classical Hodgkin lymphoma

**Figure 1:**
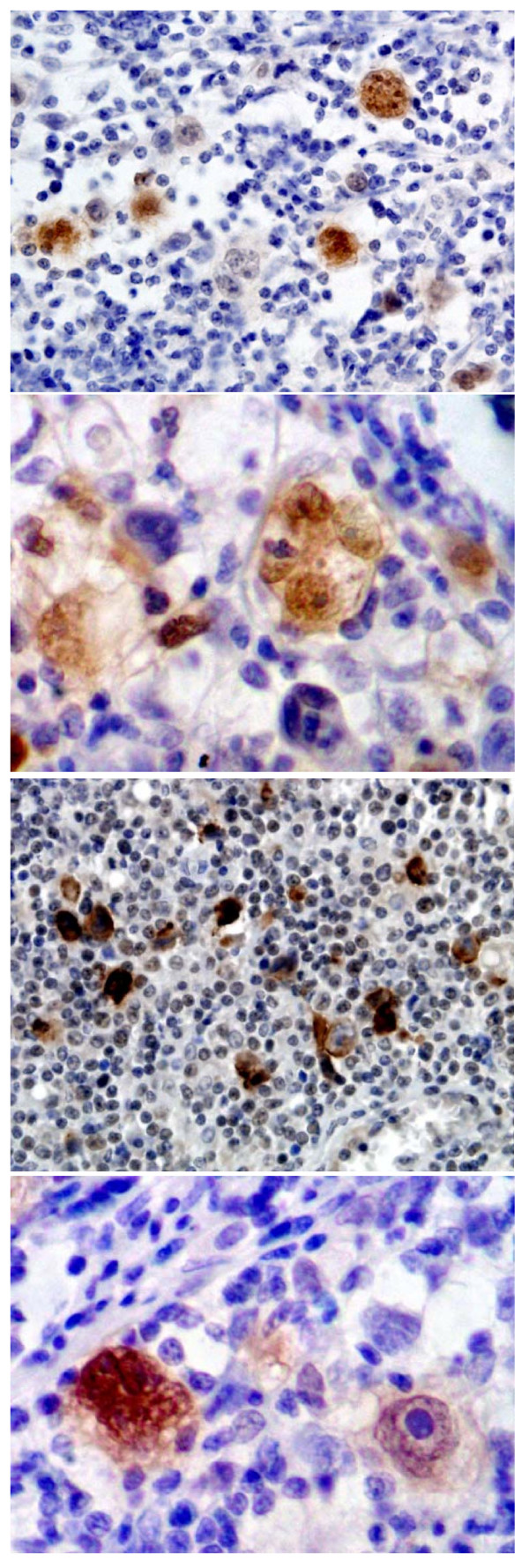
**Expression of ID2, EBV-LMP1 and P16(INK4A) in classical Hodgkin lymphoma**. ID2 expression in HRS cells (A: 200×; B: 400×); EBV-LMP1 expression in HRS cells (C: 200×); P16(INK4A) expression in HRS cells (D: 400×).

ID2 protein was expressed positively in 83.3% (50 out of 60) of the classical Hodgkin lymphomas (Table 1). It was strongly positive in both cytoplasm and nucleus of the HRS cells (Figure 1A, B), and also positive in plasma cells or lymphoplasmacytoid cells but negative or only minimally positive in reactive B and T lymphocytes in the background of Hodgkin lymphoma. It was noted that some HRS cells did not express ID2. In normal lymphatic tissues, ID2 expression was mainly restricted to a few GC and interfollicular cells. Based on morphology, the ID2-positive cells were dendritic cells and macrophages. Fibroblasts and endotheliocytes were positive for ID2 as well as smooth muscle cells of blood vessels. Eight of ten B-cell lymphomas and three of eight T-cell lymphomas were stained positively for ID2 (Table 1).

**Table 1:** Expression of ID2, EBV-LMP1 and P16(INK4A) in classical Hodgkin lymphoma

Lymphoma	n	ID2	EBV-LMP1	P16(INK4A)
**Classical HL**				
Nodular sclerosis	29	25	24	22
Mixed cullularity	11	10	9	7
Lymphocyte-rich	16	12	14	11
Lymphocyte-depleted	4	3	4	3
				
**Non-HL**				
DLBCL	5	4	2	3
MALT	4	3	0	1
Burkitt's	1	1	1	1
ALCL	2	2	1	1
AILT	2	1	2	1
NK/T(nasal type)	2	0	2	1
Peripheral TCL	2	0	0	1
				
**Non-lymphomatous**				
Tonsilla	2	2*	0	0
Lymph nodes	10	2*	0	0

HL: Hodgkin lymphoma

DLBCL: diffuse large B cell lymphoma

MALT: extranodal marginal zone B-cell lymphoma of mucosa-associated lymphoid tissue

Burkitt's: Burkitt's lymphoma

ALCL: anaplastic large cell lymphoma

AILT: angioimmunoblastic T-cell lymphoma

*: restricted to a few GC and interfollicular cells

EBV-LMP1 protein was expressed in 85.0% (51 out of 60) of this group of classical Hodgkin lymphomas (Table 1). EBV-LMP1 was noted in the HRS cells, highlighting them from the complex background of lymphocytes (Figure 1C) although positive staining could also be found minimally or occasionally in the surrounding lymphocytes. Staining was mainly nuclear and infrequently cytoplasmic. However, some HRS cells were negative for EBV-LMP1 staining. Three of ten B-cell lymphomas and five of eight T-cell lymphomas, but none of the ten reactive lymph nodes were stained positively for EBV-LMP1 (Table 1).

P16(INK4A) protein was noted in 71.7% (43 out of 60) of the classical Hodgkin lymphomas by immunohistochemistry (Table 1). P16(INK4A) was overexpressed mainly in the nuclei of Hodgkin and Reed-Sternberg (HRS) cells (Figure 1D). Some Hodgkin cells showed some cytoplasmic staining of P16(INK4A) protein as well as its nuclear staining. Mummified HRS cells were often negative for P16(INK4A) protein. Many of the HRS cells with a very prominent nucleolus were positive for P16(INK4A). However, some HRS cells were negative, distinct from the positive cells nearby. Neither B nor T lymphocytes in the background of the lymphoma or other tissues showed positive staining for P16(INK4A) protein, although histiocytes were weakly positive. There was no P16(INK4A) staining in the lymphocytes, plasma cells and eosinophils. In the control tissues and reactive lymph nodes, histiocytic lineage cells including macrophages, Langerhans cells, and dendritic cells were weakly positive for P16(INK4A). In the non-Hodgkin lymphomas (B-cell and T-cell lymphomas), P16(INK4A) protein was found in five out of ten B-cell lymphomas and four out of eight T-cell lymphomas, but none of lymphocytes in the reactive lymph nodes were positive (Table 1).

### The association between ID2 and EBV-LMP1 or P16(INK4A)

Under the microscope, ID2 was detected in most EBV-LMP1 positive HRS cells, and there was a positive correlation between the two markers (4.400 ± 2.980 *vs *4.267 ± 2.842, Rho = 0.946, *P *= 0.0001). However, ID2 was not correlated with P16(INK4A) in this group of Hodgkin lymphomas (4.400 ± 2.980 *vs *3.567 ± 2.557, Rho = -0.120, *P *= 0.191). It was also noted that HRS cells positive for EBV-LMP1 were negative for P16(INK4A) and *vice versa *in the same field on serial sections. The inverse correlation between two proteins was significant (4.267 ± 2.842 *vs *3.567 ± 2.557, Rho = -0.206, *P *= 0.024). Among nine EBV-LMP1 totally negative tumours, four was positive for P16(INK4A) and also four positive for ID2. Of 51 EBV-LMP1 positive cases, 12 was negative for P16(INK4A) and 9 negative for ID2 completely.

## Discussion

ID2 can inactivate E2A and perhaps PAX5, and recurrent chromosomal gains of the ID2 gene might contribute to the aberrant expression [19,20]. We show here that ID2 is not detectable in normal B cells but is strongly expressed in HRS cells of 83.3% of Chinese classical Hodgkin lymphoma (50/60). These findings are in agreement with those of a previous work from Germany [14], although our ratio is slightly lower than in that report (42/42) [14]. These results suggest a possible role for ID2 in repressing B-cell-specific gene expression by inactivation of E2A and PAX5 in Hodgkin lymphoma. EBV positivity rates in classical Hodgkin lymphoma vary considerably across the world [18,21-25] likely reflecting in part different pathogenesis. In our series, using EBV-LMP1 immunohistochemistry, we noted EBV positivity in 85.0% of cases. Other series have used in situ hybridization for EBV early RNAs (EBER) as well as EBV-LMP1 immunostaining which may explain some of the differences in EBV detection rates. In our study, ID2 expression was expressed in the vast majority of classical Hodgkin lymphoma (83.3%) so it may be a potentially useful marker for identifying the HRS cell in classical Hodgkin lymphoma, independent of EBV status. In this group of Hodgkin lymphomas from China, P16(INK4A) was also overexpressed. It was found in 71.7% of the classical Hodgkin lymphomas, again, much higher than in the Malasian (49.2%) [18] but in agreement with that study it was inversely correlated with EBV-LMP1 (*P *< 0.05). The phenomenon supports the notion that the pathogenesis of Hodgkin lymphoma is different from that of other tumors that lose P16(INK4A). However, there may be an association with other effects of ID2 on HRS cells such as inactivation of the anti-proliferative function of Rb, which could indirectly induce overexpression of P16(INK4A)[26,27].

Taken together, ID2, EBV-LMP1 and P16(INK4A) were highly expressed in HRS cells of classical Hodgkin lymphoma, distinguishing the HRS cells from the background lymphocytes.

## Conclusion

Our study here suggests that combined with cellular morphology, the three proteins could be considered as potential adjunct markers to identify HRS cells in diagnosis of classical Hodgkin lymphoma.

## Competing interests

The authors declare that they have no competing interests.

## Authors' contributions

ZP carried out the design, analysis of pathology and drafted the manuscript. LY carried out sample collecting and co-ordination. LL performed the immunohistochemical staining. ZM carried out slide sectioning. All authors read and approved the manuscript.

## Pre-publication history

The pre-publication history for this paper can be accessed here:


